# A rare case of successful treatment of peritoneal dialysis patient with *Serratia marcescens* peritonitis without catheter removal: case report and literature review

**DOI:** 10.3389/fcimb.2024.1373036

**Published:** 2024-05-30

**Authors:** Ruizhi Xie, Ying Ling, Yaru Huang, Lulu Qin, Kun Bao, Xindong Qin

**Affiliations:** ^1^The Second Clinical Medical College of Guangzhou University of Chinese Medicine, Guangzhou, China; ^2^The Sixth Clinical Medical College, Shenzhen Hospital (Futian) of Guangzhou University of Chinese Medicine, Shenzhen, Guangdong, China; ^3^The College of Basic Medical Sciences of Guangzhou University of Chinese Medicine, Guangzhou, China; ^4^Nephrology Department, Guangdong Provincial Hospital of Chinese Medicine, Guangzhou, China; ^5^State Key Laboratory of Dampness Syndrome of Chinese Medicine, The Second Affiliated Hospital of Guangzhou University of Chinese Medicine, Guangzhou, China; ^6^Guangdong Provincial Key Laboratory of Chinese Medicine for Prevention and Treatment of Refractory Chronic Diseases, Guangzhou, China

**Keywords:** case report, *Serratia marcescens*, peritoneal dialysis, peritonitis, infection route, treatment

## Abstract

Serratia marcescens, as a Gram-negative opportunistic pathogen, is a rare cause of peritonitis and has worse clinical outcomes than Gram-positive peritonitis. In this case report, we describe a case of Serratia marcescens associated peritonitis that was successfully cured without catheter removal. A 40-year-old male patient with peritoneal dialysis who worked in the catering industry was admitted to the hospital for 16 hours after the discovery of cloudy peritoneal dialysate and abdominal pain. Ceftazidime and cefazolin sodium were immediately given intravenously as an empirical antibiotic regimen. After detecting Serratia marcescens in the peritoneal diasate culture, the treatment was switched to ceftazidime and levofloxacin. The routine examination of peritoneal dialysate showed a significant decrease in white blood cells, the peritoneal dialysate became clear, and the peritoneal dialysis catheter was retained. The patient was treated for 2 weeks and treated with oral antibiotics for 1 week. It is necessary to further strengthen the hygiene of work environment to prevent Serratia marcescens infection in peritoneal dialysis patients. We recommend that patients with Serratia marcescens associated peritonitis should be treated with a combination of antibiotics as early as possible empirically, and at the same time, the peritoneal dialysis fluid culture should be improved, and the antibiotic regimen should be timely adjusted according to the drug sensitivity results. For patients with clinical symptoms for more than 3 days, considering the strong virulence of Serratia marcescens, whether to use meropenem directly or not can provide a reference for clinical decision-making. Further clinical studies are needed to achieve more precise anti-infective treatment.

## 1 Introduction

Chronic kidney disease (CKD) is a worldwide concern. An observational study based on the KDOQI criteria showed that the prevalence of CKD stage 5 was about 13.4% (Hill et al., 2016; Evans et al., 2022). The progression of chronic kidney disease remains an important cause of reduced quality of life and premature death. To maintain the normal metabolic homeostasis of the human body, there are a considerable number of people who cannot obtain kidney donors under the condition of limited donor kidneys. Peritoneal dialysis or hemodialysis before waiting for kidney transplantation is still a common renal replacement therapy for patients with end-stage renal disease (Bonomini et al., 2020); Some studies have compared hemodialysis (HD) with peritoneal dialysis (PD), and although the evidence is not conclusive, the two methods are generally considered to be equivalent (Wong et al., 2018; Bellasi et al., 2020). Hemodialysis is used more frequently in the world, but peritoneal dialysis offers obvious technical advantages, such as continuous solute and fluid removal, loose restrictions on water, sodium, potassium and phosphorus, less cardiovascular impact, residual renal function preservation, lower mortality, flexible operation, and better quality of life (Mehrotra et al., 2016). Use has increased in recent years and is relatively more common in younger age groups (Saran et al., 2015).

Peritoneal dialysis-related peritonitis is the most common infectious complication in peritoneal dialysis patients, manifesting mainly as abdominal pain, fever, nausea and vomiting (Kokubu et al., 2020), It may lead to serious consequences such as dialysis failure, spread of infection and even death. A common reason for conversion to hemodialysis during peritoneal dialysis is that a single severe episode of peritonitis or multiple episodes of peritonitis often result in decreased peritoneal ultrafiltration capacity (Szeto and Li, 2019; Khan, 2023; Piarulli et al., 2023). Gram-positive cocci such as Staphylococcus and Enterococcus and multi-drug resistant bacteria are the common pathogens of peritonitis. Serratia marcescens, a Gram-negative conditionally pathogenic bacterium, is a rare cause of peritonitis with a worse clinical outcome than Gram-positive peritonitis (Szeto et al., 2005; Kang et al., 2013). We report a case of peritoneal dialysis in a patient with Serratia marcescens peritonitis that was successfully cured without catheter removal after treatment with multiple antibiotics.

## 2 Case presentation

The patient, male, 40 years old, who worked in the cooking industry, was admitted to the hospital due to “abdominal pain for 16 hours with cloudy peritoneal dialysate fluid”. The patient was diagnosed with IgA nephropathy by renal puncture 5 years ago and had stage 5 secondary chronic kidney disease. Peritoneal dialysis catheterization was performed. Then continuous ambulatory peritoneal dialysis (CAPD) has been on the shelf for more than 5 years. The current peritoneal dialysis regimen is 1.5% glucose dialysate 2 L×4 bags, and the last bag remaining 2 L. The patient had no previous history of peritonitis episodes. The main symptoms on admission were persistent severe abdominal pain and peritoneal dialysis fluid turbidity. After emergency treatment, the patient was admitted to the Department of Nephrology of Guangdong Provincial Hospital of Chinese Medicine because of no obvious analgesic effect.

**Figure 1:**
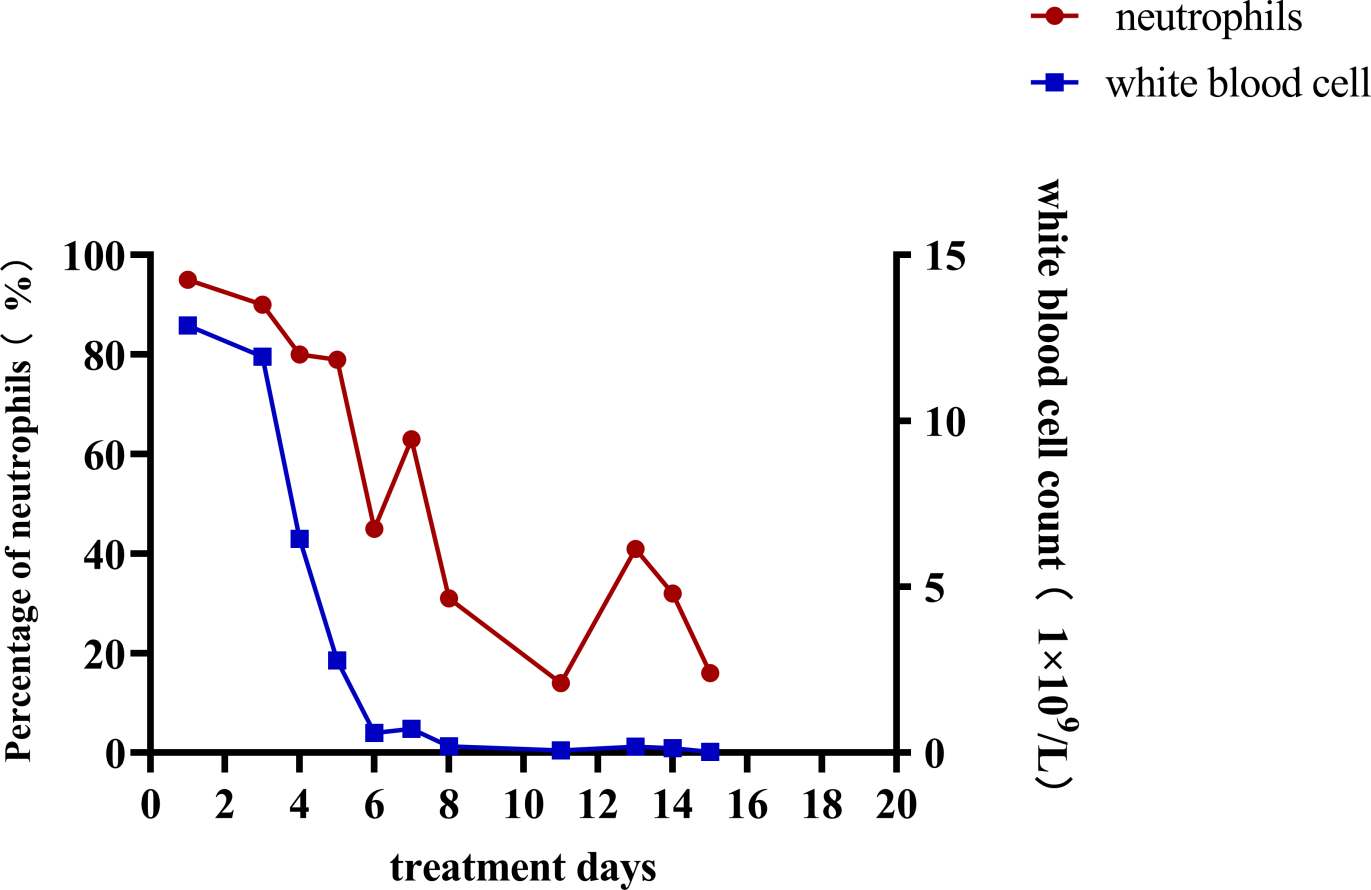
White blood cell count and percentage of neutrophils in peritoneal dialysis effluent.

After admission, the relevant examination showed that the white blood cell count in the peritoneal dialysate was 12878*10^6/L, and the neutrophils were 95%. The white blood cell count was 11.98*10^9/L, and the procalcitonin was 41.66 ng/ml. After admission, peritoneal lavage was performed immediately, and ceftazidime (2.0g/qd) and cefazolin sodium (1.0g/qd) were given empirically for anti-infection. Intravenous piperacillin sodium and tazobactam (2.25g/q12h) was given at the same time because infection could not be ruled out. The white blood cell count in the peritoneal dialysate was 11934*10^6/L, and the neutrophil count was 90%. Two days later, the pathogen was identified by culture as Serratia marcescens (**Table 1**), and peritoneal dialysis-associated peritonitis was confirmed as Serratia marcescens infection. According to the drug sensitivity results, the antibiotics were changed to ceftazidime (2.0g/qd) and levofloxacin (0.25g/qd). After adjusting antibiotics, the routine review of abdominal dialysis fluid showed that the white blood cells decreased significantly to 6445*10^6/L, and the procalcitonin decreased to 25.31 ng/ml. Although the patient still had abdominal pain, the abdominal permeate solution was clearer than the previous one. Considering the treatment is effective, the current treatment plan should be continued. Within 2 weeks of administration, the patient’s clinical symptoms improved significantly, abdominal pain was relieved, and the peritoneal permeation test showed that the white blood cell count was 26*10^6/L, and the neutrophils were 16% (**Figure 1**). The peritoneal permeation culture results also turned negative. After discharge, the patient underwent consolidation therapy with oral cefixime (0.1g/q12h) and levofloxacin (0.25g/qd). No recurrence of symptoms was observed until the last follow-up. Applying the data in **Table 2**, an attempt was made to review the patient’s entire treatment regimen based on the entire treatment cycle during the patient’s hospitalization.

**Table 1:** Microbiology results from patient’s peritoneal fluid.

Peritoneal Fluid Culture		
Culture: Serratia Marcescens		
Antibiotics	MIC/KB	Sensitivity
Amoxicillin/clavulanic acid	8μg/ml	R
Tigecycline	1μg/ml	S
Levofloxacin	<=0.12μg/ml	S
Ertapenem	<=0.12μg/ml	S
Cefoxitin	16μg/ml	R
Ceftazidime	0.25μg/ml	S
Cefoperazone/Sulbactam	<=8μg/ml	S
Imipenem	24mm	S
Cotrimoxazole	<=20μg/ml	S
Amikacin	<=2μg/ml	S
Cefuroxime axetil	16μg/ml	R
Cefepime	<=0.12μg/ml	S
ceftriaxone	<=0.25μg/ml	S
Cefuroxime sodium	16μg/ml	R

R, Resistance; S, Sensitive.

**Table 2:** Antibiotics used during peritonitis.

Time	Dosage regimen
Day1-Day2	IP: ceftazidime(2.0g/qd) and cefazolin(1.0g/qd) + IV: piperacillin sodium tazobactam(2.25g/q12h)
Day3-Day15	IP: ceftazidime(2.0g/qd) and levofloxacin(0.25g/qd) + IV: cefoperazone sodium and sulbactam sodium(1.5g/q12h)
Day16-Day22	PO: cefixime(0.1g/q12h) and levofloxacin(0.25g/qd)

IP, intraperitoneal; IV, intravenous; PO, oral.

## 3 Discussion

Peritoneal dialysis-related peritonitis refers to the onset of peritonitis at any time after peritoneal dialysis treatment, which is a common complication of peritoneal dialysis. The diagnosis is mainly based on clinical manifestations, white blood cell count in peritoneal dialysate, and ascites culture results. According to the updated peritonitis guidelines of the International Society for Peritoneal Dialysis (ISPD) (Li et al., 2022), The diagnosis should meet at least two of the following criteria: 1. Clinical manifestations consistent with peritonitis, such as abdominal pain and cloudy peritoneal dialysate; 2. Peritoneal dialysate leukocyte count >100/ul or >0.1x10^9/L, polymorphonuclear cell count >50%; 3. The result of dialysate culture was positive. Because the possible causes of abdominal pain include some abdominal diseases and the turbidity of peritoneal dialysate may also be caused by non-infectious causes, the routine ascites test and the results of peritoneal dialysate culture are especially important for the diagnosis of peritoneal dialysis-related peritonitis. Even the results of dialysate culture and drug sensitivity can provide a basis for the selection of subsequent antibiotics. In this case, on the basis of the history collection, clinical examination results, and the culture of peritoneal dialysate fluid for Serratia marcescens (**Figure 1**; **Table 1**), it can be clearly defined as peritoneal dialysis-related peritonitis, rather than catheter-related peritonitis, gut-derived peritonitis, prePD peritonitis, PD-related peritonitis, or PD catheter-related peritonitis.

Using “serratia marcescens”, “peritoneal dialysis” and “peritonitis” as the common search terms, we searched the PubMed database published before October 2023, and a total of 12 related articles were retrieved. The types of literature collected included retrospective analysis and case reports. At present, there are relatively few reports on Serratia marcescens induced peritoneal dialysis-related peritonitis. Epidemiological data were obtained from the only 12 cases reported at present, excluding the cases with incomplete data, it was found that 60% of the cases were male and 49% were female, and the average age was 48 years old, the youngest age was 22 years old and the oldest age was 79 years old. Only 5 of these cases were successfully cured without catheter removal. The statistical results are shown in **Table 3**. Based on the literature and case review, the following points are proposed.

**Table 3:** Review of previously reported cases of Peritoneal dialysis without catheter removal and successful cure of Serratia marcescens infection peritonitis from Library building to 2023.10.

Author	Year	Age (years)/gender	Duration of clinical symptoms before treatment	Treatment		Route of infection	Treatment days
				Empiric antibiotic therapy	According to the results of peritoneal dialysate culture		
Hortling et al. L (Hortling and Sipilä, 1984)	1984	ND	ND	/	Aztreonam	ND	ND
Grabe DW et al (Grabe et al., 1997)	1997	47/M	4d	Vancomycin and gentamicin(iv)	gentamycin and ceftizoxime(ip)	ND	14 d
Bhave P et al (Bhave et al., 2016)	2016	67/M	ND	cephazolin and gentamycin(ip)	Meropenem(ip)	hospital infection	21d
Sarihan I et al (Sarihan et al., 2017)	2017	57/F	ND	Cefazoline(ip)+ ciprofloxacin(po)	gentamycin(ip)+ ciprofloxacin(po)	poor home environment and hygienic conditions	21d
Ning Y et al (Yang and Li, 2021)	2020	79/M	3d	cefazolin and ceftazidime(ip)	Levofloxacin, then cefoperazone sodium and sulbactam sodium, meropenem, and finally amikacin(ip)	ND	29d
Current case	2023	40/M	16h	ceftazidime and cefazolin(ip)+ piperacillin sodium tazobactam(iv)	ceftazidime and levofloxacin(ip) + cefoperazone sodium and sulbactam sodium(iv), then cefixime and levofloxacin(po)	work environment	22d

M, male; F, female; IP, intraperitoneal; IV, intravenous; PO, oral; ND, not date; D, day; /, no.

Serratia marcescens is a saprophytic gram-negative bacterium that is widely found in water, soil, insects, animals, and plants, and some strains produce a red pigment, prodigiosin. In 1951, Professor Wheat first reported nosocomial infection caused by Serratia marcescens (Wheat et al., 1951). Until now it had been considered a non-pathogenic bacterium (Hegazy et al., 2021). The pathogenesis of Serratia marcescens is attributed to a variety of virulence factors, biofilm formation, motility and alfalfa pigment production, as well as the production of various extracellular enzymes (such as nucleases, hemolysins, proteases and lipases) (Van Houdt et al., 2007). Infections caused by Serratia marcescens have been reported with increasing frequency since 1960 (Dodson, 1968). Serratia belongs to Enterobacterales with high genetic plasticity. It can cause a wide range of nosocomial infections (Lazarus et al., 2021). It is more likely to occur in neonatal intensive care unit and surgical ward. Transient trolley cross-transmission by health care workers, transient hand contamination for cross-transmission, contaminated water sources, and breast milk appear to be possible routes of transmission in the neonatal intensive care unit (Chokephaibulkit et al., 2002; Milisavljevic et al., 2004; Giles et al., 2006; Bechmann et al., 2023; Bourdin et al., 2023). Another case-control study concluded that total parenteral nutrition solutions may constitute a possible route of introduction of microorganisms into the neonatal intensive care unit (Maltezou et al., 2012). Data suggest that in surgical wards, intravenous fluids may be the route of transmission and that medical staff play an important role in transmitting infection (Chokephaibulkit et al., 2002), causing nosocomial infection outbreaks. Causing nosocomial infection outbreaks. At present, there are relatively few reports on the induction of peritoneal dialysis-related peritonitis by Serratia marcescens. Many predisposing factors are related to previous use of antibiotics, immunosuppression, diabetes, renal failure, steroid use, underlying gastrointestinal lesions and malignant tumors (Barraclough et al., 2010). We collected retrospective cases to try to analyze the infection route of the pathogen. One patient was exposed to the hospital environment during peritoneal dialysis (Bhave et al., 2016), which strengthened the evidence related to nosocomial infection. The evidence related to nosocomial infection was strengthened. Another female (Sarihan et al., 2017)presented with abdominal pain and nausea after 5 months of automated peritoneal dialysis (APD), which she shared with 11 other socioeconomic family members in a room with poor sanitation. Our case was a patient with continuous ambulatory peritoneal dialysis. After inquiring about the medical history, it was found that the patient was a cook and had a history of diarrhea for 4 days before the onset of the disease. It was speculated that the patient’s working environment was relatively complex and she was easily exposed to a variety of pathogenic bacteria.

Serratia marcescens is multi-drug resistant to a variety of antibiotics, including penicillins, cephalosporins, tetracycline, macrolides, nitrofurantoin, colistin (Zivkovic Zaric et al., 2023), The drug susceptibility results of our case showed that it was resistant to Amoxicillin/clavulanic acid and Cefuroxime axetil (**Table 1**), which was consistent with the literature. In particular, it can cause catheter-related infections, especially in biofilm colonies, which can further increase resistance to antibiotics (Ray et al., 2017). Therefore, special attention should be paid to the placement of intravenous catheters, abdominal catheters or urinary catheters, and mechanical ventilation devices (Buttinelli et al., 2023). The clinical prognosis of peritonitis associated with gram-negative bacteria is worse than that associated with gram-positive bacteria.

Currently, there are no guidelines for the treatment of S. marcescens. Third-generation cephalosporins, such as cefotaxime and ceftazidime, are the mainstay for the treatment of S. marcescens infections. S. marcescens is susceptible to producing AmpC enzyme and has the gene encoding AmpC on its chromosome (Tamma et al., 2019), which is co-regulated by AmpD, AmpE, AmpG, and AmpR genes (Chow et al., 1991). However, Enterobacteriaceae produce heterogeneous levels of AmpC, with S. marcescens strains expressing up to 10-fold lower levels than inhibited E. cloacae or C. freundi isolates (Power et al., 2006; Kohlmann et al., 2018), and different β-lactam drugs have different ability to induce AmpC activity. Broad-spectrum cephalosporins such as cefotaxime, ceftazidime, ceftriaxone, and cefepime are weak inducers of this enzyme and thus maintain relatively stable levels against Ampc-induced bacteria (Jacoby, 2009). β-lactamase inhibitors (βLIs) have the most potent βLIs activity for AmpC production (Pogue et al., 2019). The EUCAST expert rules recommend that the use of cephalosporin-susceptible Enterobacteriaceae, Seratella, etc. as monotherapy is discouraged because of the risk of selection for resistance. The site of infection, the density of the bacterial population, the level of drugs available at the site of infection, and the clinical condition of the patient should be taken into account in treatment. Broad-spectrum β-lactamase-stable cephalosporins were combined with β-lactamase inhibitors (Kohlmann et al., 2018). Because fluoroquinolones do not have a β-lactam ring, they are suitable for the treatment of mild to moderate infections caused by AmpC (Tamma et al., 2019).

Due to the rarity of peritonitis caused by Serratia marcescens, there is a lack of clinical large sample analysis, and the relevant literature is mostly based on case reports. The differences in cure probabilities between the infecting organism and antibiotic regimens suggest the need for further investigation of microbial-specific therapies. Based on the resistance of Serratia marcescus, clinicians will start empirical intraperitoneal injection of antibiotics as soon as possible as the main means of treatment. In our case, the combination of ceftazidime and cefazolin sodium was first used to cover gram-positive and Gram-positive bacteria when the drug susceptibility results were not confirmed, and then the medication was adjusted according to the drug susceptibility results of peritoneal fluid (**Table 2**).

In addition, it has been suggested that the risk of treatment failure increases by 5.5% for every 1- hour delay in antibiotic treatment (Muthucumarana et al., 2016). Another Japanese study showed that a delay of 24 hours or more in antibiotic administration was associated with a threefold increased risk of PD catheter removal compared with the time to definitive dialysate results (Oki et al., 2021). So far, no optimal antibiotic regimen has been clearly determined. Empirical intraperitoneal antibiotics mainly cover Gram-positive and Gram-negative bacteria, and the antibiotic regimen and subsequent treatment decisions can be further changed based on the results of peritoneal dialytic culture. In our case and the case reported by Yang and Li (2021), empirical anti-infective therapy with cefazolin combined with ceftazidime was started immediately after the diagnosis of peritonitis. Although the two cases were not successfully removed and cured, the careful comparison showed that the antibiotic regimens of Yang and Li were changed several times after the results of drug sensitivity were confirmed. Finally, the clinical symptoms were relieved after 10 days of anti-infective treatment with intraperitoneal injection of meropenem. For the empirical treatment of PD-related peritonitis, current guidelines advocate the use of cephalosporins in the abdomen, such as cefepime or a combination of cefazolin and cefixime (Li et al., 2010). However, the increasing number of multi-drug resistant bacteria has shifted our attention to antibacterial drugs with a wider range of activities. Carbapenems, especially meropenem, have more and more successful experience in the treatment of PD-related peritonitis reported in the literature (Van Ende et al., 2010; Vlaar et al., 2013). It has been reported that the intraperitoneal application of meropenem in the treatment of peritonitis in PD patients is feasible. In the case of the investigator, the patient was admitted to the hospital for 3 days because of abdominal pain and the discovery of cloudy peritoneal dialysate. It is reasonable to assume that the time of initiation of antibiotics has some influence on the subsequent administration of antibiotics. Although meropenem as a first-line drug is not the most appropriate choice in terms of microbiology, due to the high virulence of Serratia marcescens, strong and effective antibiotics can still be considered first if it is growing for a long time.

## 4 Conclusion

Serratia marcescens is a very rare pathogen that causes peritoneal associated peritonitis. It has strong virulence, which is easy to lead to adverse consequences such as peritoneal dialysis tube removal, replacement of dialysis methods and even death. The main routes of infection were nosocomial infection, family environment and work environment. It is reminded that medical staff must strengthen hand hygiene in the hospital, and lay ambulatory peritoneal dialysis patients also need to pay attention to hand hygiene, the family and work environment to avoid infection. In the course of clinical treatment, if there are symptoms of turbidity of peritoneal dialysate and abdominal pain, it is necessary to improve etiological culture immediately, and adjust antibiotic types according to drug sensitivity results; If the patient’s clinical symptoms have been admitted to hospital for more than 3 days, and the etiological culture results support peritoneal dialysis associated peritonitis caused by Serratia marcescens, meropenem may be directly used to achieve the purpose of infection control as soon as possible, which still needs to be verified by further basic and clinical studies.

## Data availability statement

The original contributions presented in the study are included in the article/supplementary material. Further inquiries can be directed to the corresponding authors.

## Ethics statement

The studies involving humans were approved by Ethics Committee of Guangdong Provincial Hospital of Chinese Medicine. The studies were conducted in accordance with the local legislation and institutional requirements. The participants provided their written informed consent to participate in this study. Written informed consent was obtained from the individual(s) for the publication of any potentially identifiable images or data included in this article.

## Author contributions

RX: Conceptualization, Data curation, Formal analysis, Investigation, Project administration, Writing – original draft, Writing – review & editing. YL: Conceptualization, Data curation, Formal analysis, Investigation, Project administration, Writing – original draft, Writing – review & editing. YH: Software, Writing – review & editing. LQ: Software, Writing – review & editing. KB: Project administration, Resources, Supervision, Writing – review & editing. XQ: Formal analysis, Investigation, Project administration, Resources, Supervision, Writing – review & editing.
